# Role of ABO Secretor Status in Mucosal Innate Immunity and H. pylori Infection

**DOI:** 10.1371/journal.ppat.0040002

**Published:** 2008-01-04

**Authors:** Sara Lindén, Jafar Mahdavi, Cristina Semino-Mora, Cara Olsen, Ingemar Carlstedt, Thomas Borén, Andre Dubois

**Affiliations:** 1 Laboratory of Gastrointestinal and Liver Studies, Digestive Diseases Division, Department of Medicine, Uniformed Services University of the Health Sciences, Bethesda, Maryland, United States of America; 2 United States Military Cancer Institute, Bethesda, Maryland, United States of America; 3 Mucosal Diseases Program, Mater Medical Research Institute, South Brisbane, Australia; 4 Division of Microbiology and Infectious Diseases, Queen's Medical Centre, Nottingham, United Kingdom; 5 Department of Preventive Medicine and Biometrics, Uniformed Services University of the Health Sciences, Bethesda, Maryland, United States of America; 6 Mucosal Biology Group, Department of Cell- and Molecular Biology, BMC, Lund University, Lund, Sweden; 7 Department of Medical Biochemistry and Biophysics, and Laboratory for Molecular Infection Medicine Sweden (MIMS), Umeå University, Umeå, Sweden; Harvard Medical School, United States of America

## Abstract

The fucosylated ABH antigens, which constitute the molecular basis for the ABO blood group system, are also expressed in salivary secretions and gastrointestinal epithelia in individuals of positive secretor status; however, the biological function of the ABO blood group system is unknown. Gastric mucosa biopsies of 41 Rhesus monkeys originating from Southern Asia were analyzed by immunohistochemistry. A majority of these animals were found to be of blood group B and weak-secretor phenotype (i.e., expressing both Lewis a and Lewis b antigens), which are also common in South Asian human populations. A selected group of ten monkeys was inoculated with Helicobacter pylori and studied for changes in gastric mucosal glycosylation during a 10-month period. We observed a loss in mucosal fucosylation and concurrent induction and time-dependent dynamics in gastric mucosal sialylation (carbohydrate marker of inflammation), which affect H. pylori adhesion targets and thus modulate host–bacterial interactions. Of particular relevance, gastric mucosal density of H. pylori, gastritis, and sialylation were all higher in secretor individuals compared to weak-secretors, the latter being apparently “protected.” These results demonstrate that the secretor status plays an intrinsic role in resistance to H. pylori infection and suggest that the fucosylated secretor ABH antigens constitute interactive members of the human and primate mucosal innate immune system.

## Author Summary

The common ABO blood group antigen system was described in the early 20th century. In addition, it has been known for 60 years that the majority of individuals also express the corresponding ABO antigens (carbohydrate identity tags) in their saliva, tears, milk, and mucus secretions in the digestive tract. To this date, however, the biological function of the ABO blood group antigens has remained an enigma. Here, we show that the great majority of Rhesus monkeys are of blood group B and weak-secretors, i.e., are similar to the human populations in South Asia from where these monkeys originate. This observation suggests that an evolutionary adaptation in digestive tract mucosal carbohydrate patterns to local environmental selection has occurred. In addition, we demonstrate that long-term infection by the “peptic ulcer bacterium” Helicobacter pylori induces mucosal carbohydrate patterns that change according to the individual secretor phenotype. The common weak-secretor monkeys were apparently “protected,” as they had stable glycosylation, lower inflammation, and lower bacterial infection load, whereas the less common secretor animals had increased levels of inflammation-associated mucosal carbohydrate patterns and a transient decrease in the ABO blood group system type of carbohydrates. These novel observations suggest that the individual ABO blood group and secretor phenotype are part of human and non-human primate innate immunity against infectious disease.

## Introduction

In turbulent systems such as the oro-gastro-intestinal tract, adaptation to local niches requires microbial adherence properties to match host receptors and thereby stabilize microbial colonization or infection. H. pylori achieved this goal by developing the BabA and SabA adhesins, which bind to the host fucosylated blood group (bg) ABO antigens (denoted the ABH antigens) and sialylated Lewis antigens, respectively [1–5]. These adhesins are relevant to H. pylori pathogenicity since BabA-positive strains are frequently present in both peptic ulceration and gastric cancer [5–8]. H. pylori infection and associated gastritis induce expression of mucosal sialylated receptors for the SabA adhesin that also attract peripheral neutrophils to local areas of inflammation [2,9]. In turn, H. pylori SabA adhesin acts as a “selectin-mimic” in mediating binding to both sialylated epithelium and to neutrophils [2,10].

**Figure 1:**
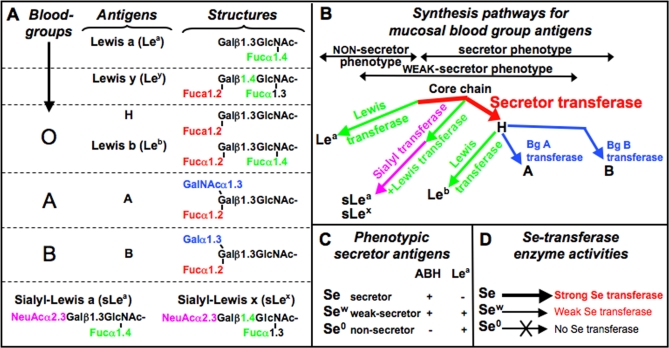
Fucosylated and Sialylated Blood Group (bg) Antigens and Associated Secretor Phenotypes (A) The α1.2-fucosylated (in red) H and Le^b^ antigens define bg O. Bg A and B antigens present additional GalNAc or Gal residues (blue), respectively. Le^y^ and Le^b^ are both difucosylated. SLe^a^ and sLe^x^ are sialylated Lewis antigens (in pink). (B) Synthesis pathways for bg antigens with corresponding Se phenotypes: Le^a^ is found in Se^0^ individuals, whereas Se^w^ individuals carry a mix of mucosal Le^a^ and Le^b^. Le^a^ is formed when the Se-transferase is inactive or weak, because Le^a^ is a “dead-end” and is not extended further. During inflammation and infection, sialyl-transferases are expressed and carbohydrate core chains become sialylated in competition with Se-fucosyltransferase. (C) The presence of ABH and Le^a^ antigens in salivary, milk, and GI tract secretions identifies individuals of Se, Se^0^, or Se^w^ phenotype. (D) In Se^w^ subjects, α1.2fucosylation is hampered by an enzymatically weak Se-transferase, whereas Se^0^ individuals lack Se-transferase activity.

The A, B, and H antigens are complex fucosylated carbohydrates expressed on erythrocytes of all individuals of blood group A, B, or O, respectively. The common denominator is the Fucα1.2-glycan presented by all three ABH antigens, because the bone marrow (from where the erythrocytes originate) express the common H-(fucosyl)transferase. Lack of fucosylated ABH antigens on erythrocytes in circulation (Bombay phenotype) is exceedingly rare. The ABH antigens are also expressed along the oro-gastro-intestinal (GI) mucosal lining in individuals of “positive secretor status” (secretor phenotype, Se) [11,12]. This is because Se individuals express the Secretor-(fucosyl)transferase, which is the enzyme that produces the Fucα1.2-glycan structure, the hallmark of ABH antigens in saliva, and gastrointestinal mucus secretions and epithelium (see Figure 1A for bg antigens). Due to the mucosal secretor-transferase, gastrointestinal epithelia of secretors express blood group O antigens [Lewis b (Le^b^) and H antigens], which can be extended by a GalNAc- or Gal-residue into bg A or B antigens, respectively [12] (Figure 1). In contrast, individuals of non-secretor phenotype, Se^0^, lack the secretor-transferase altogether, and make the shorter Lewis a antigen (Le^a^) [13] (Figure 1). A third and most recently described human secretor phenotype, the weak-secretor phenotype, Se^w^, is characterized by expression of both Le^a^ and Le^b^ antigens. The composite of Le^a^ and Le^b^ antigens is the consequence of a weak (mutated) form of the secretor transferase [11] (Figure 1B and Figure 1C).

A protective mucus layer comprised of mucin glycoproteins carrying multitudes of carbohydrate structures covers the mucosal surfaces. This glyco-mucus layer exhibits rapid turnover and is shed into the GI tract lumen together with scavenged and aggregated secretions, desquamated cells, and microorganisms. Mucins from both humans and Rhesus monkeys efficiently bind H. pylori via fucosylated carbohydrates [14,15]. Thus, fucosylated host secretions such as mucins, saliva, and milk, inhibit adherence of H. pylori and other microbial pathogens to the mucosal cell surfaces [16–18].

A majority of Caucasians (80%) are secretors, whereas 20% of them are non-secretors, and weak-secretor individuals are rare or not yet discovered. In contrast, weak-secretor individuals are common among Chinese, Japanese, Polynesians, Australian aborigines, and African-Americans [11]. The skewed prevalence in secretor phenotypes suggests selection in response to specific types of infections or other environmental conditions. Indeed, Se^0^ individuals are more at risk for urinary tract infections [19,20]; Se^0^ subjects also express higher inflammatory reactivity and sialylated host antigens to H. pylori infection, which may explain the higher prevalence of peptic ulcer disease observed in those subjects [21–23]. In contrast, Se^0^ individuals are less likely to develop Norwalk virus–induced acute gastroenteritis due to the lack of mucosal Se-dependent ABH antigens that mediate mucosal adherence of virus particles in Se subjects [24].

Here, the dynamics of mucosal responses to H. pylori infection was studied in Rhesus monkeys because this animal is subject to natural H. pylori infection and exhibits human-like patterns of gastric glycosylation [15,25–27]. Furthermore, the complete description of the macaque genome is available for this important primate model [28]. After determining the ABO blood groups and secretor phenotypes of 41 Rhesus monkeys, we inoculated virulent H. pylori strains to representative secretor groups. During the persistent H. pylori infection that ensued, gastric mucosal fucosylation transiently decreased and sialylation reciprocally increased. *In vivo H. pylori* density, inflammation, and *in vitro* adherence of H. pylori to sialylated antigens were all lower among weak-secretors (the common Rhesus monkey phenotype) compared to regular secretors.

We propose that mucosal glycosylation on GI cell surfaces and secretions as determined by secretor status together influence the course of H. pylori infection as part of the primate innate immunity.

## Results

### Rhesus Monkey ABO Blood Groups and Secretor Phenotypes

Immunostaining of gastric biopsies from 41 Rhesus monkeys without H. pylori infection demonstrated that 28 animals (68%) were of bg B and 13 (32%) were of bg AB. Thus, mucosal bg B antigen was expressed in all 41 monkeys. The positive immunostaining of bg B and A antigens in the gastric epithelium also demonstrates that all animals were secretors (Se) of ABH-antigens and, hence, that non-secretors (Se^0^ that lack ABH antigens in the GI mucosal lining) were not represented in this large group of monkeys (Figure 1). In addition, 34/41 (83%) animals expressed both Le^a^ and Le^b^, an antigen combination that, in humans, is regarded as the characteristics of the weak-secretor phenotype (Se^w^). Only seven monkeys did not express mucosal Le^a^ and they were identified as regular Se. Thus, Se^w^ status with the weak form of secretor transferase appears to be the predominant secretor phenotype in the Rhesus monkey.

### Secretor Phenotype Affects H. pylori Infection Density and Gastritis

A total of ten animals, 3/7 Se (bg B) and 7/34 Se^w^ (bg B, Le^a+^ Le^b+^) animals, were selected for H. pylori inoculation experiments, and two virulent CagA and VacA positive H. pylori strains became predominant in most animals after a few months [29]. We determined that these two strains could bind both ABH and sLe^a/x^ antigens (see Materials and Methods). Persistent high-grade infection was observed in 9/10 monkeys and only animal 86D02 (Se^w^) demonstrated low-grade infection (H. pylori score of 1, gastritis score ranging from 1 to 2, but negative cultures, Table S1).

**Figure 2:**
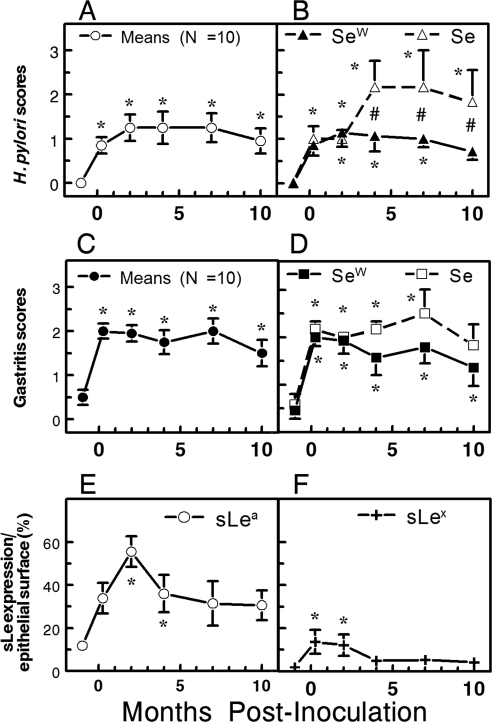
Infection Density, Gastritis, and Mucosal Sialylation in Proximal Stomach (Antrum), in Response to H. pylori Infection (Means ± SEM) The figure illustrates the time course of H. pylori infection density scores in biopsies from the ten monkeys (A) and in three Se and seven Se^w^ individuals following inoculation (B). Also illustrated is the time course of gastritis scores in biopsies from the ten monkeys (C) and from the three Se and seven Se^w^ individuals following inoculation (D). Finally, the mean percentages of sLe^a^ positive (E) and sLe^x^ positive surface epithelium (F) are shown in the ten monkeys following inoculation. * Illustrates significant (*P* <0.05) difference from pre-inoculation value and # illustrates significant (*P* <0.05) difference between Se and Se^w^.

H. pylori infection and associated gastritis developed similarly in the antrum (Figure 2A and 2C) and in the corpus (Figure S2A and S2C) starting at day 7 after infection. In both regions of the stomach, *H. pylori in vivo* density score was 2-fold higher in Se than in the dominant Se^w^ phenotype from 4 to 10 months after inoculation (Figures 2B and S2B, *P* <0.05). Consistent with the higher infection load**,** secretors had increased levels of gastritis compared to weak-secretors but the difference was significant only in the corpus (Figures 2D and S2D). Interestingly, corpus gastritis scores increased significantly compared to pre-inoculation levels only in Se monkeys but not in Se^w^ animals (Figure S2D). Finally, H. pylori infection density and gastritis were strongly correlated (Table 1A).

**Table 1:**
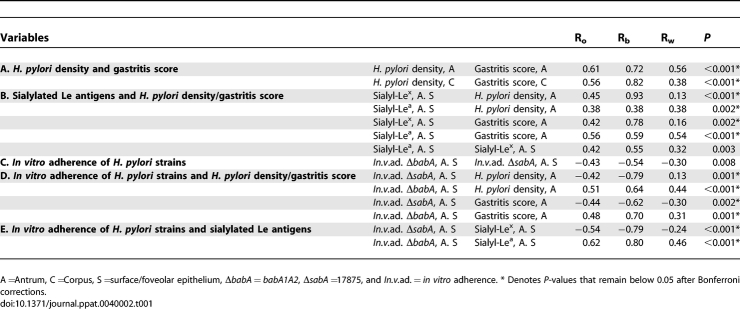
Correlation Coefficients for Parameters That Were Found to be Statistically Significant (See “Statistics” in Materials and Methods and in Protocol S1)

### Time-Dependent Suppression of Fucosylated Blood Group Antigens during Infection

**Figure 3:**
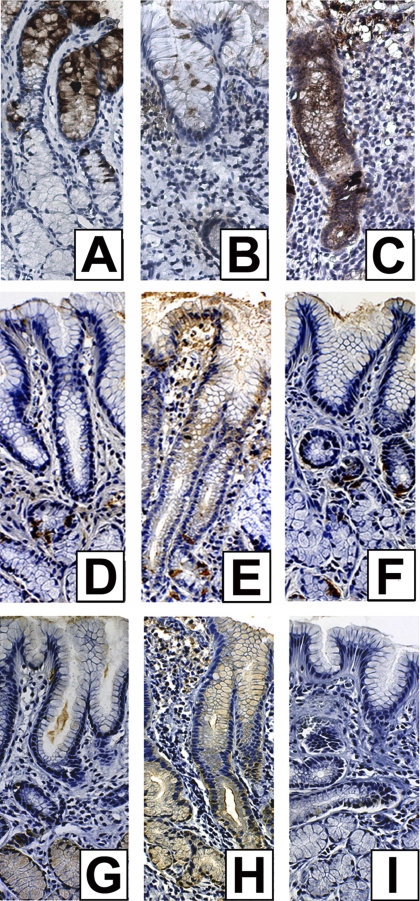
Dynamic Reciprocity in Expression of Fucosylated and Sialylated Antigens in Gastric Mucosa during H. pylori Infection Le^b^ expression in monkey 8PZ at pre-inoculation (A), 1 week post-inoculation (B), and 2 months post inoculation (C). Thus, gastric mucosal fucosylation was initially strong (A), decreased from 1 to 4 weeks post-inoculation (B), returned to basal level at 2 months (C), and stayed at basal level until 10 months (not shown). The figure also illustrates the expression of sialyl-Le^a^ (D, E, and F) and sialyl-Le^x^ (G, H, and I) in monkey 8PZ at pre-inoculation (D and G), 2 months (E and H), and 10 months post-inoculation (F and I). Thus, gastric mucosal sialylation increased during early infection (not shown), peaked at 2 months (E and H), and returned to pre-inoculation levels at 10 months post-inoculation (F and I).

In all seven Se^w^ monkeys, both Le^b^ and Le^a^ antigens were expressed in surface epithelium before experimental infection (Figure 3A). At 1–4 weeks after inoculation, Le^b^ (Figure 3B) and/or Le^a^ expression (data not shown) transiently decreased in 5/7 monkeys**.** However, by 2–4 months, expression of Le^b^/Le^a^ returned to pre-inoculation levels or higher (Figure 3C). Similarly, the fucosylated Lewis y (Le^y^) antigen expressed in the gastric glands of both Se and Se^w^ non-infected animals initially decreased in response to inoculation, and then returned to baseline in 8/10 monkeys (data not shown). Thus, H. pylori infection causes general suppression of fucosylation in the gastric mucosa, as reflected by the alterations in the different Le^a^, Le^b^, and Le^y^ antigens (this series of fucosylated Lewis antigens are described in Figure 1A).

### Expression of Sialylated Lewis Antigens in Dynamic Response to Infection and Gastritis

The mean percentage of sialyl-Lewis a (sLe^a^) and sialyl-Lewis x (sLe^x^) positive surface epithelial cells rapidly increased in all monkeys with established H. pylori infection (Figure 2E and 2F in antrum and in Figure S2E and S2F in corpus). Sialyl expression is also illustrated in Figure 3D–3I. Expression of sLe^a^ was stronger than that of sLe^x^, and the percentage of sLe^a^ positive cells rapidly increased 4-fold within a week of inoculation. Thus, a majority of surface epithelial cells (60%) express strong sLe^a^ at 2 months both in antrum and corpus (Figures 2E and S2E). Similarly, expression of sLe^x^ was strongest at 2 months (Figure 2F). Expression of sialylated antigens later decreased in seven monkeys (Figures 2E and S2E and also illustrated in Figure 3F and 3I). Importantly, sLe^a^ and sLe^x^ expression in surface epithelium of antrum correlated with both H. pylori density and gastritis scores (see Table 1B).

### Stable Expression of Gastric Type of Mucin Core Proteins during Infection

The observed shifts in glycosylation were not due to pre-dysplastic alterations of the gastric mucosa such as intestinal metaplasia, because the spatial distribution of expression patterns for the two common gastric mucins MUC5AC and MUC6 (Figure S3) were unchanged during the 10-month period of experimental infection, with no aberrant MUC2 or atypical sulfo-mucins being detected (data not shown).

### The *In Vitro* Adherence Patterns Revealed by BabA and SabA Mutants Paralleled the Changes in Mucosal Fucosylation and Sialylation Observed during Infection *In Vivo*

The significant correlation between H. pylori density and gastritis and the similarity in time course (Figures 2A, 2C, S2A, and S2C) suggest that the higher level of mucosal inflammation is a consequence of the higher infection load. Because of the exclusive binding properties of BabA (for fucosylated antigens) and SabA (for sialylated antigens) mediated binding properties [3,30], H. pylori mutants were applied as lectin-like tools in an *in vitro* adherence assay to functionally map detailed shifts specific for secretor-dependent mucosal fucosylation and sialylation induced by H. pylori infection and the associated inflammatory responses. The Δ*sabA* (BabA+) mutant bound fucosylated, secretor-dependent ABH/Le^b^ antigens (but not the shorter Le^a^ or sialylated antigens), whereas the Δ*babA* (SabA+) mutant bound inflammation-associated sialylated (but not fucosylated) antigens [2,5,31]. Representative adherence patterns of binding to Rhesus monkey biopsies by the Δ*sabA* (BabA+) and the Δ*babA* (SabA+) mutants are shown in Figure S1. In histo-tissue sections of gastric mucosa, the Δ*sabA* (BabA+) mutant binds to the foveolar epithelium cell surfaces and intra cellular mucins and, in addition, to the secreted fucosylated mucus layer. These binding tests demonstrate that BabA positive bacteria co-localize with the MUC5AC mucin (compare Figure S3A and S3C). By comparison, the Δ*babA* (SabA+) mutant does not bind to the surface epithelium of non-infected Rhesus monkey gastric mucosa, and SabA-mediated binding instead colocalizes with the sialylated MUC6 mucin expressed in the deeper located glandular region (compare Figures S3B and S3D). However, when the mucosa during infection has responded with expression of inflammation-associated antigens, the Δ*babA* (SabA+) mutant binds to the sialylated foveolar epithelium and mucus layer (illustrated in Figure S1).

**Figure 4:**
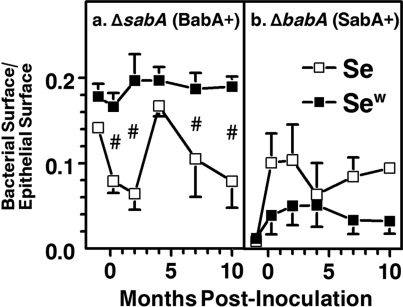
Time Course of Gastric Mucosal Fucosylation and Sialylation following Inoculation of H. pylori to Rhesus Monkeys as Determined by the *In Vitro* Adherence Assay The figure illustrates that inoculation of H. pylori induces a strong, time-dependent, increase in mucosal fucosylation and a concurrent suppression of inflammation-associated sialylation in Se monkeys (**□**), whereas changes are milder in individuals of Se^w^ phenotype (). The changes in the glycosylation pattern were followed by use of *in vitro* adherence analyses and fluorescent H. pylori bacterial cells as glycosylation-specific lectin tools. Because the H. pylori Δ*sabA* (BabA+) and Δ*babA* (SabA+) mutants bind primarily to fucosylated and sialylated structures, respectively, the time-dependent changes measured in terms of *in vitro* adherence reflect parallel changes in surface epithelium fucosylation and sialylation. In vitro adherence mediated by the BabA adhesin (in Figure S1) identifies the mucosal expression patterns of secretor-dependent fucosylation of ABH and Le^b^ antigens (A), whereas adherence mediated by the SabA adhesin reveals mucosal sialylation (B). Importantly, only *in vitro* adherence to surface epithelium is shown, because it is fucosylated by the Se-transferase. From each of 120 biopsies, ten gastric pit regions were acquired for digital analysis (i.e., a total of 1,200 mucosal zones were analyzed) [31]. # Illustrates significant (*P* <0.05) differences between Se and Se^w^ subjects at the corresponding times.

Importantly, *in vivo*, H. pylori binds to the intact apical cell surfaces and secreted extracellular mucins in mucus, whereas by the *in vitro* adherence assay and use of histo-tissue sections, the H. pylori bacterial cells also bind to intracellular mucins that have been exposed by histo sectioning of the mucosal cells and tissue. Thus, the *in vitro* adherence method provides an unique opportunity to investigate time-dependent changes in secretor-dependent mucosal glycosylation that occurs during H. pylori infection, i.e., using small pinch biopsies collected at regular intervals during an extended study period and without sacrificing the animals. In the basal state, the BabA-positive Δ*sabA* mutant adhered *in vitro* to the non-inflamed, non-infected gastric mucosa of all ten Rhesus monkeys (see Figures 4A and S3C), in accordance with the mucosal expression of secretor-dependent bg B antigen in all monkeys. Following H. pylori inoculation, the dynamics of glycosylation and inflammation were tightly correlated (see Table 1), as revealed by the rapid and transient decrease in BabA-mediated *in vitro* adherence that paralleled the expression patterns of fucosylated antigens (Compare Figure 4A with Figure 3A–3C).

Expression of secretor-dependent mucosal fucosylation involved in H. pylori adhesion remained strong and robust throughout the 10-month observation period in Se^w^ subjects compared to the rapid initial decrease in fucosylation in Se individuals, as revealed by BabA-mediated *in vitro* adherence to fucosylated bg antigens (Figure 4A). In addition, BabA-mediated *in vitro* adherence to fucosylated mucosa was 1.7 times higher in Se^w^ than in Se monkeys (*P* <0.001) and inversely correlated with *in vivo H. pylori* density, gastritis, and sLe^x^ expression (see Tables 1D and 1E, respectively). Thus, Se^w^ with strong and robust expression of gastric fucosylation have both lower *H. pylori* infection density and less gastritis compared to Se individuals (Figures 4A, 2B, and 2D).

The strong fucosylation in Se^w^ monkeys, as revealed by the robust BabA-mediated *in vitro* adherence, reflects the high density of fucosylated mucins in the Se^w^ gastric mucosa. Indeed, gastric mucins purified from healthy, non-infected Rhesus monkey bind H. pylori primarily via fucosylated structures (Figure S3E), similarly to human mucins binding to H. pylori [4,14].

In contrast to BabA-mediated adherence, *in vitro* adherence to sialylated glycoconjugates by the SabA positive Δ*babA* mutant was absent in the surface epithelium of healthy, uninfected mucosa. However, SabA-mediated adherence rapidly increased in response to inoculation, thus demonstrating induction of mucosal sialylation. SabA-mediated *in vitro* adherence to the surface epithelium correlated with both *in vivo H. pylori* density and gastritis (Table 1D) and with expression of the inflammation-associated sLe^x^ and sLe^a^ antigens (Table 1E). Furthermore, SabA-mediated *in vitro* adherence to sialylated glycoconjugates was 1.9-fold higher in Se than in Se^w^ monkeys (Figure 4B). The rapid induction of SabA-mediated binding to the sialylated surface epithelium of the gastric mucosa (Figure 4B) demonstrates that Se monkeys react with stronger inflammatory response and higher recruitment of inflammatory cells, i.e., gastritis, whereas the Se^w^ animals with low grade infection and inflammation provides sparse mucosal sialylation and only modest recruitment of inflammatory cells. Fucosylation and sialylation levels follow opposite dynamics during the full 10-month observation period (fucosylation in Figures 4A and 3A–3C; sialylation in Figures 4B and 3D–3I; Table 1C). Thus, Se^w^ individuals are more robust in mucosal fucosylation and balanced in sialylation, which confers lower inflammatory level, lower gastritis, and lower H. pylori infection density as compared to Se individuals (Figures 2B, 2D, S2B, and S2D).

## Discussion

The present study demonstrates that Se^w^ individuals have robust mucosal fucosylation and lower mucosal inflammatory and sialylation responses to experimental H. pylori infection. Therefore, Se^w^ monkeys would be expected to better tolerate persistent infections and to be more prevalent in regions with high incidence of this type of infections. Such a selection for specific blood group phenotypes is strongly suggested by our observation that all 41 macaques included in the study express blood group B antigens, especially since the blood group B is the least common worldwide of the ABO phenotypes. Interestingly, the ancestors of most macaques used in the present study originated from Northern India, which is the region with highest worldwide prevalence of bg B phenotype in humans [32,33]. The recent Rhesus macaque genome annotation revealed that the Indian and Chinese groups diverged some 160,000 years ago [34]. Interestingly, all the ABO blood groups are represented among macaques that moved to Southeast Asia/Thailand [35], a region in which bg B is lower in humans [32]. The human and macaque paralleled demographic selection for high prevalence of Se^w^ and bg B phenotype may result from selection by endemic infectious disease [36] as the two species often suffer from the same infectious diseases. Of particular relevance for the Indian subcontinent, bg B individuals are less likely to become infected by Vibrio cholera [37]. In addition, phylogenetic analysis suggests that bg B arose several times and independently from bg A, indicating that the genes of these blood group transferases are prone to convergent evolution [38].

The ABO blood groups were discovered over a century ago [39], chemically described 50 year ago [40,41], and cloned almost 20 years ago [42]. Similarly, the secretor system was described 60 years ago [43], blood group antigens were characterized from human intestine over 30 years ago [44] and the Se transferase was cloned in 1995 [13], but the biological and functional role of the ABO system has remained an enigma [45]. Here, we show that secretor phenotype determines the dynamics of mucosal glycosylation in response to H. pylori infection and conditions the nature of the host response. Thus, H. pylori infection is associated with an increase in sialylated mucosal antigens and a concurrent decrease in fucosylated mucosal antigens. The loss of fucosylation during acute H. pylori infection is probably a consequence of the fast induction in expression of inflammation-associated sialyl-transferases and the resulting competition for carbohydrate chains by glycosyl (sialyl and fucosyl) transferases (Figure 1B). Competition between fucosyl- and sialyl-transferases for the same carbohydrate core chains was demonstrated by competition experiments where di-saccharides suppressed both sialylation and formation of selectin (endothelial cell adhesion) ligands on cancer cells [46]**.** The present series of results also reveals that, in contrast to Se monkeys, Se^w^ individuals maintain strong and robust expression of fucosylated mucosal ABH antigens during H. pylori infection (Figure 4A). Interestingly, intestinal mucosal glycosylation also becomes fucosylated in response to establishment of conventional bacterial flora in gnotobiotic mice [47].

The combined results suggest that mucosal fucosylation could be a mixed blessing for *H. pylori.* Indeed, large mucin molecules with fucosylated high-affinity binding sites for BabA could be exploited by H. pylori as *in vivo* binding sites, but they may also act as scouts of the host glycan innate immunity system. Thus, the mucosal fucosylation represents a protective scavenger factor that reduces infection density, especially since H. pylori infection also increases gastric mucus secretion [48]. The present demonstration that mucosal fucosylation in response to H. pylori infection reduces bacterial density and associated inflammation and, in particular, impacts on infection in Se^w^ monkeys due to stronger mucosal fucosylation phenotype, strongly suggests that ABH secretor-dependent mucosal glycosylation modulates innate immunity responses and may contribute to variable risk of gastric disease.

## Materials and Methods

### Experimental animals.

The experiments were conducted according to the “Guide for the Care and Use of Laboratory Animals” [49]. All procedures involving animals were reviewed and approved by the USUHS animal care and use committee. The ancestors of 37 animals had been captured in India, while four were of mixed Chinese and Indian origin. The localization of Lewis antigens in these monkeys before H. pylori inoculation has been investigated [15]. H. pylori and H. heilmannii infection was eradicated in all monkeys 6 months before inoculation [29]. The ten male monkeys that were inoculated all originated from India and were 4–13 years old (mean 7.1). F754, 86D02, T4C, 8V5, F436, 82A49, and 8PZ animals were Se^w^, and 86D06, E6C, and 85D08 were Se.

### Inoculation strains and cultures.

Seven low-pass H. pylori strains were cultured, characterized (five were CagA+ and two were CagA-) (see Text S1), and inoculated to monkeys as reported [29]. Their binding properties were analyzed by RIA [31]: J170, J254, J166, and J258 bound both sLe^x^ and Le^b^, J282 bound Le^b^, and J178 bound sLe^x^.

### Immunohistochemistry.

Genta-stained sections were used to determine H. pylori density scores and the Sydney system was used to determine gastritis scores [29]. Sulfo-mucins were detected with high-iron diamine stain [17]. Immunohistochemistry was performed as described [15] (see Text S1). For quantitative histochemistry, tissue sections were stained simultaneously. Non-sialylated carbohydrate structures were quantified by visual estimation of intensity, whereas a program by J. Czege, USUHS, was used for sialylated antigens. The surface/foveolar epithelium, lamina propria and glands were separately outlined and the percentage area stained was average from three fields of view.

### H. pylori adherence *in vitro.*

The 17875/Le^b^-mutant was referred to as the Δ*sabA* (BabA^+^) mutant or “BabA-positive mutant”, and the isogenic 17875*babA1::kan babA2::cam* deletion mutant was referred to as Δ*babA* (SabA^+^) mutant or “SabA-positive-mutant [2,5]. In vitro adherence was digitally quantified in a total of 1,200 mucosal zones (120 biopsies, ten pits/biopsy) [31] (see Text S1).

### Statistics.

Data are reported as means ± SEM. Changes over time within animals were compared with a mixed-effects ANOVA model corresponding to a repeated-measures ANOVA model with time as a within-subject factor and secretor status as a between-subjects factor. Dunnett's post-hoc test was used to compare the average at each time point to the average before inoculation. For each pair of variables, three correlation coefficients were calculated by ANOVA with random effects (see Protocol S1/Statistics).

## Supporting Information

Figure S1 BabA Adhesin-Mediated Binding of H. pylori to Fucosylated ABO/Le^b^ Blood Group Antigens and SabA Adhesin-Mediated Binding of H. pylori to Sialylated and Inflammation-Associated Antigens in Primate Gastric Mucosa (3.9 MB DOC)

Figure S2 Infection Density, Gastritis and Mucosal Sialylation in the Proximal Stomach in Response to H. pylori Infection (1.0 MB DOC)

Figure S3 Rhesus Monkey Gastric Mucins (550 KB DOC)

Protocol S1 Supporting Protocols 1. Reagents
2. *H. pylori* culture
3. Mucin isolation and analyses of blood group glycosylation
4. *H. pylori* binding to mucins in ELISA
5. Statistics
(34 KB DOC)

Table S1 Individual Values of Gastritis Scores, *H. pylori In Vivo* Density, and Antral and Body sLe^x^ and sLe^a^ Staining for Antrum and Body, and Antral Mucosal Fucosylation and Sialylation Patterns Revealed by *In Vitro* Adherence Mediated by BabA and SabA, Respectively (266 KB DOC)

Text S1 Supporting References (46 KB DOC)

## Abbreviations

ABH antigens: fucosylated blood group (bg) ABO antigens
BabA: blood group antigen–binding adhesin; bg blood group
GI: gastro-intestinal
Leb: Lewis b
SabA: sialic acid–binding adhesin
sLe^x^: sialyl-Le^x^
Se: secretor phenotype
Se^w^: weak-secretor phenotype
Se^0^: non-secretor phenotype
