# Supplementary material for: Role of ABO Secretor Status in Mucosal Innate Immunity and H. pylori Infection
Source: PLoS Pathog. 2008 Jan 4;4(1):e2. doi: 10.1371/journal.ppat.0040002 (PMC2174967; doi:10.1371/journal.ppat.0040002)
Supplement: Supplementary file 1 [file ppat.0040002.sd001.doc]

**Supporting Information**

**Protocols**

**Reagents.** polyclonal antibodies against MUC6 (LUM6-3) (1) and MUC2 (LUM2-3) (2); a monoclonal antibody against MUC5AC (45M1) (Sigma-Aldrich, Steinheim, Germany); monoclonal antibodies against Le^b^ (clone 2-25LE) and Le^a^ (clone 7LE, kind gifts from Dr. J. Bara, INSERM, France), Le^a^ (BG5), Le^b^ (BG6), and Le^y^ (BG8) (Signet, Dedham, MA), bg A (clone 81 FR 2.2), B (clone 3E7) and H (clone 92FR-A2) antigens from Dako A/S, Copenhagen, Denmark, sLe^x^ (clone KM93) and sLe^a^ (clone 1H4, Seikagaku America, Ijamsville, MD); biotinylated goat anti-mouse and goat anti-rabbit sera and Strept AB complex/HRP (Vector Laboratories, Burlingame, CA); and Biotin blocking system (Dako A/S, Copenhagen, Denmark).

***H. pylori* Culture*.*** Gastric biopsies harvested at the time of each endoscopy were cultured on *Campylobacter* chocolate blood agar plates supplemented with trimethoprim, vancomycin, amphotericin B and polymyxin B and *H. pylori* isolates were characterized and subcultured as described [1]

**Mucin isolation and analyses of blood group glycosylation.** Rhesus monkey mucins were isolated by isopycnic density gradient centrifugation as previously described [2]. For ELISA, samples were coated onto microtiter plates overnight. The microtiter plates were washed 3 times in 0.15 M NaCl, 0.05% Tween, and 5 mM sodium phosphate buffer, pH 7.4 (washing buffer), between all ensuing steps. Unbound sites were blocked with 0.5% bovine serum albumin in washing buffer (blocking buffer) for 1 hour. The microtiter plates were incubated with the primary antibody diluted in blocking buffer for 1 hour. Plates were then incubated with alkaline phosphatase conjugated secondary antibody, either goat anti-mouse IgG antibodies, goat anti-mouse IgM, streptavidin, or swine anti-rabbit serum in blocking buffer, for 1 hour. Bound secondary antibody was visualized using p-nitrophenyl phosphate as a substrate (2 mg/mL in 0.5 mM MgCl2 and 1 mM diethanolamine-HCl buffer, pH 9.8). Absorbance at 405 nm was measured after 1 hour.

***H. pylori* binding to mucins in ELISA*.*** For *H. pylori* adhesion experiments, mucin aliquots were coated onto microtiter plates at room temperature in humidified chambers overnight. The microtiter plates were washed 3 times in washing buffer between all ensuing steps. Unbound sites were blocked with 1% Boehringer blocking reagent for ELISA containing 0.05% Tween and 10 mM citric acid for 1 hour. The microtiter plates were incubated with a suspension of biotinylated bacteria OD600 = 0.15 diluted 100 times in 1% Blocking reagent for ELISA (Boehringer Mannheim, Mannheim, Germany) containing 0.05% Tween for 2 hours at 37°C with orbital shaking. Then the plates were incubated with alkaline phosphatase-conjugated streptavidin and bound streptavidin was visualized using nitrophenyl phosphate (2 mg/ml in 1 M diethanolamine-HCl buffer, pH 9.8, containing 0.5 mM MgCl_2_). Absorbance at 405 nm was measured after 1 hour.

**Statistics.** Changes over time within animals were compared with a mixed-effects ANOVA model corresponding to a repeated-measures ANOVA model with week as a within-subject factor. Data are reported as mean ± SEM. Dunnett's post-hoc test was used to compare the average at each time point to the average at week 0. For each pair of variables, three correlation coefficients were calculated by ANOVA with random effects. The within-subjects correlation (R_w_) measures the extent to which an increase in one variable over time is associated with an increase in the second variable over time [3]. The between-subjects correlation (R_b_) measures the extent to which monkeys with high values of one variable have high values of a second variable. R_b_ is essentially the correlation between the means of a certain parameter for each monkey [4]. The overall correlation (R_o_) measures the extent to which high values for one variable are associated with high values for a second variable, across all monkeys and time points, and thus R_o_ combines aspects of both R_w_ and R_b_. Correlations were calculated on data from 10 monkeys at 6 time points and the significance of the correlations (p) was determined after taking into account the lack of independence of multiple observations on the same monkey. p <0.05 was considered statistically significant. Although some variables did not appear to follow a normal distribution, examination of residual plots showed that the assumptions of the significance test for correlation were met. Statistical analyses were performed with STATA 7.0 for Windows (StatCorp, College Station, TX), except for Dunnett's post-hoc test, which was performed using SAS (R) version 9.0 (SAS Institute Inc., Cary, NC).
