# Supplementary material for: Role of ABO Secretor Status in Mucosal Innate Immunity and H. pylori Infection
Source: PLoS Pathog. 2008 Jan 4;4(1):e2. doi: 10.1371/journal.ppat.0040002 (PMC2174967; doi:10.1371/journal.ppat.0040002)
Supplement: Supplementary file 2 [file ppat.0040002.sd002.doc]

**Supporting Information**

**References**

**1.** Dubois A, Berg DE, Fiala N, Heman-Ackah LM, Perez-Perez GI, Blaser MJ (1998) Cure of Helicobacter pylori infection by omeprazole-clarithromycin-based therapy in non-human primates. J Gastroenterol 33: 18-22.

**2.**	Nordman H, Davies JR, Lindell G, de Bolos C, Real F, Carlstedt I. (2002) Gastric MUC5AC and MUC6 are large oligomeric mucins that differ in size, glycosylation and tissue distribution*. Biochem* J. 364:191-200.).

**3.**	Bland JM, Altman DG (1995) Calculating correlation coefficients with repeated observations. Part 1- -Correlation within subjects. British Medical Journal 310: 446.

**4.**	Bland JM, Altman DG (1995) Calculating correlation coefficients with repeated observations: Part 2- -Correlation between subjects. British Medical Journal 310: 633.

**5.** [Aspholm M, Kalia A, Ruhl S, Schedin S, Arnqvist A, Linden S, Sjostrom R, Gerhard M, Semino-Mora C, Dubois A, Unemo M, Danielsson D, Teneberg S, Lee WK, Berg DE, Boren T.](http://www.ncbi.nlm.nih.gov/sites/entrez?Db=pubmed&Cmd=ShowDetailView&TermToSearch=17132512&ordinalpos=1&itool=EntrezSystem2.PEntrez.Pubmed.Pubmed_ResultsPanel.Pubmed_RVDocSum) (2006) Helicobacter pylori adhesion to carbohydrates. Methods Enzymol. 417:293-339.
