# Supplementary material for: Role of ABO Secretor Status in Mucosal Innate Immunity and H. pylori Infection
Source: PLoS Pathog. 2008 Jan 4;4(1):e2. doi: 10.1371/journal.ppat.0040002 (PMC2174967; doi:10.1371/journal.ppat.0040002)
Supplement: Supplementary file 3 [file ppat.0040002.sg001.doc]

**Supporting Information**

**Figure S1.** BabA adhesin mediated binding of *H. pylori* to fucosylated ABO/Leb blood group antigens (A) and, SabA adhesin mediated binding of *H. pylori* to sialylated and inflammation associated antigens (B), respectively, in primate gastric mucosa. As a complement to immunohistochemistry analyses, the dynamic changes in terms of secretor-dependent fucosylation and sialylation patterns in gastric mucosa in response to infection and inflammation were analyzed by use of bacterial *in vitro* adherence. By this technique, fluorescently labeled *H. pylori* cells are overlaid onto deparaffinized, rehydrated histo-tissue sections of gastric mucosa, and the levels of adherence are digitally analyzed [5]. *In vitro* adherence by the BabA positive strain 17875/Le^b^ was used as a probe for secretor-dependent fucosylated blood group antigens and the isogenic SabA positive mutant was used to probe specifically for sialylated antigens. The BabA positive strain (A) adhered to the surface epithelium and mucus layer of all monkeys (left arrow), but not to the glandular region (right arrow). By comparison, the SabA positive mutant (B) adhered to inflammation-associated sialylated antigens both in the surface epithelium (left arrow), and in the glandular region (right arrow).
