# Supplementary material for: Role of ABO Secretor Status in Mucosal Innate Immunity and H. pylori Infection
Source: PLoS Pathog. 2008 Jan 4;4(1):e2. doi: 10.1371/journal.ppat.0040002 (PMC2174967; doi:10.1371/journal.ppat.0040002)
Supplement: Supplementary file 4 [file ppat.0040002.sg003.doc]

**Supporting Information**

**Figure S3**. **Rhesus monkey gastric mucins.** The first barrier the pathogen encounters is the highly hydrated mucus gel that covers the mucosal surface and protects the epithelial cells against chemical, enzymatic, microbial and mechanical insult. This mucus layer is not static but has a high turnover and is continuously shed into the gastric lumen. In the stomach, the mucus layer is formed by the MUC5AC (panel A) and MUC6 mucins (panel B), which are produced by the surface and gland cells respectively. The mucin producing epithelial cells are highly specialized with the cell filled with mucins waiting to get released, and the nucleus, endoplasmic reticulum and Golgi pushed close to the basolateral membrane (panel A and B). In the *in vitro* adherence assay, the bacteria adhere to intracellular mucins (arrow in panel C) and to the secreted mucus layer. In contrast, during *in vivo* conditions, the bacterial cells come in contact only with secreted mucins. By *in vitro* adherence tests and based on studies of gastric biopsies of *H. pylori* uninfected stomach, the BabA adhesin mediates *H. pylori* binding to MUC5AC mucin (panel C) and to the MUC6 mucin via the SabA adhesin (panel D). In panel E, fucosylated and sialylated structures mediate binding of *H. pylori* to Rhesus monkey gastric mucins isolated by CsCl density-gradient centrifugation in 4 M GuHCl. The gradients were emptied from the tubes and fractions analyzed for density, MUC5AC, MUC6, the histo blood group structures A, B, Le^a^ and Le^b^. Furthermore, binding was analyzed with wild type strain CCUG17875 carrying both the BabA and SabA adhesins (black bar), as well as with the isogenic ΔBabA deletion mutant that express the SabA adhesin (white bar) and a strain expressing neither the BabA nor the SabA adhesin. The figure shows a summary of binding to the fraction containing the highest amount of mucins (density 1.4 mg/ml) from gastric mucosa from a healthy Rhesus monkey of histo (secreted) blood group B, i.e. a Secretor individual. No binding was detected by the *H. pylori* strain lacking both the BabA and SabA adhesins.
