# Supplementary material for: Role of ABO Secretor Status in Mucosal Innate Immunity and H. pylori Infection
Source: PLoS Pathog. 2008 Jan 4;4(1):e2. doi: 10.1371/journal.ppat.0040002 (PMC2174967; doi:10.1371/journal.ppat.0040002)
Supplement: Supplementary file 5 [file ppat.0040002.st001.doc]

**Supporting Information**

**Table 1**: Individual Values of Gastritis Scores, *H. pylori* *In Vivo* Density, Antral and Body sLe^x^ and sLe^a^ Staining for Antrum and Body, and Antral Mucosal Fucosylation and Sialylation Patterns Revealed by *In Vitro* Adherence Mediated by BabA and SabA, respectively.

| **Antrum**  Time | ID | Gastritis  score | *H.pylori*  density  score | % sLe^x^ staining in surface | % sLe^a^ staining in surface | *In vitro* binding by *H. pylori* SabA | *In vitro* binding by *H. pylori* BabA |
| --- | --- | --- | --- | --- | --- | --- | --- |
| Pre-inoculation | 82A49 | 0.50 | 0.00 | 0.6 | 28.6 | 0.014 | 0.227 |
|  | 85D08 | 0.25 | 0.00 | 1.0 | 0.7 | 0.007 | 0.155 |
|  | 86D02 | 0.00 | 0.00 | 0.1 | 25.7 | 0.001 | 0.177 |
|  | 86D06 | 1.00 | 0.00 | 0.2 | 0.7 | 0.002 | 0.145 |
|  | 8PZ | 0.75 | 0.00 | 5.7 | 5.1 | 0.053 | 0.160 |
|  | 8V5 | 1.00 | 0.00 | 0.0 | 10.1 | 0.011 | 0.172 |
|  | E6C | 0.50 | 0.00 | 0.2 | 9.4 | 0.028 | 0.130 |
|  | F436 | 1.00 | 0.00 | 0.8 | 12.3 | 0.003 | 0.140 |
|  | F754 | 0.00 | 0.00 | 4.1 | 17.8 | 0.009 | 0.218 |
|  | T4C | 0.00 | 0.00 | 7.0 | 7.5 | 0.001 | 0.193 |
| 1 week | 82A49 | 2.00 | 1.00 | 10.1 | 74.6 | 0.061 | 0.219 |
|  | 85D08 | 2.00 | 1.00 | 33.4 | 31.7 | 0.134 | 0.102 |
|  | 86D02 | 2.00 | 1.00 | 0.3 | 12.1 | 0.001 | 0.213 |
|  | 86D06 | 2.00 | 0.50 | 0.1 | 24.7 | 0.030 | 0.082 |
|  | 8PZ | 2.50 | 1.00 | 26.8 | 27.7 | 0.139 | 0.123 |
|  | 8V5 | 1.00 | 0.00 | 0.2 | 29.3 | 0.002 | 0.133 |
|  | E6C | 2.50 | 1.50 | 43.4 | 64.4 | 0.145 | 0.017 |
|  | F436 | 2.00 | 0.50 | 8.6 | 25.6 | 0.040 | 0.163 |
|  | F754 | 2.00 | 0.50 | 0.2 | 14.5 | 0.001 | 0.160 |
|  | T4C | 2.50 | 2.00 |  |  |  |  |
| 2 months | 82A49 | 2.00 | 1.00 | 11.2 | 58.0 | 0.119 | 0.282 |
|  | 85D08 | 2.00 | 1.00 | 16.3 | 37.2 | 0.024 | 0.062 |
|  | 86D02 | 2.00 | 1.00 | 4.5 | 17.8 | 0.003 | 0.241 |
|  | 86D06 | 2.00 | 1.00 | 3.2 |  | 0.164 | 0.028 |
|  | 8PZ | 2.50 | 3.00 | 40.1 | 64.5 | 0.133 | 0.076 |
|  | 8V5 | 1.00 | 1.00 | 0.0 | 62.0 | 0.093 | 0.250 |
|  | E6C | 2.00 | 1.00 | 41.3 | 69.9 | 0.121 | 0.146 |
|  | F436 | 2.00 | 0.50 | 2.7 | 33.8 | 0.003 | 0.107 |
|  | F754 | 1.00 | 0.50 | 0.4 | 72.7 | 0.004 | 0.260 |
|  | T4C | 3.00 | 1.00 | 1.8 | 84.2 | 0.003 | 0.157 |
| 4 months | 82A49 | 2.00 | 0.00 | 0.0 | 69.3 | 0.111 | 0.218 |
|  | 85D08 | 2.00 | 3.00 | 3.4 | 30.2 | 0.044 | 0.182 |
|  | 86D02 | 1.00 | 1.00 | 1.6 | 2.0 | 0.005 | 0.213 |
|  | 86D06 | 2.00 | 1.00 | 1.3 | 21.0 | 0.011 | 0.178 |
|  | 8PZ | 3.00 | 3.00 | 16.6 | 83.4 | 0.182 | 0.117 |
|  | 8V5 | 0.00 | 1.00 | 0.0 | 22.9 | 0.066 | 0.215 |
|  | E6C | 2.50 | 2.50 | 18.4 | 55.4 | 0.144 | 0.110 |
|  | F436 | 1.00 | 1.00 | 5.0 | 16.6 | 0.001 | 0.170 |
|  | F754 | 2.00 | 0.50 | 1.0 | 52.0 | 0.009 | 0.229 |
|  | T4C | 2.00 | 1.00 | 1.9 | 6.8 | 0.009 | 0.199 |
| 7 months | 82A49 | 3.00 | 0.50 | 0.4 | 84.4 | 0.121 | 0.200 |
|  | 85D08 | 3.00 | 3.00 | 4.0 | 18.8 | 0.081 | 0.118 |
|  | 86D02 | 1.00 | 1.00 | 3.8 | 7.2 | 0.000 | 0.220 |
|  | 86D06 | 1.50 | 0.50 | 2.9 | 9.3 | 0.054 | 0.187 |
|  | 8PZ | 3.00 | 1.00 | 21.6 | 68.8 | 0.069 | 0.092 |
|  | 8V5 | 1.00 | 0.50 | 0.0 | 10.0 | 0.032 | 0.238 |
|  | E6C | 3.00 | 3.00 | 12.9 | 80.4 | 0.121 | 0.002 |
|  | F436 | 1.50 | 1.00 | 4.7 | 4.2 | 0.004 | 0.177 |
|  | F754 | 1.00 | 2.00 | 2.7 | 8.3 | 0.001 | 0.214 |
|  | T4C | 2.00 | 1.00 | 0.6 | 23.2 | 0.030 | 0.199 |
| 10 months | 82A49 | 2.00 | 1.00 | 0.0 | 66.1 | 0.118 | 0.211 |
|  | 85D08 | 2.00 | 2.00 | 12.5 | 18.1 | 0.099 | 0.074 |
|  | 86D02 | 1.00 | 0.50 | 1.0 | 47.6 | 0.002 | 0.197 |
|  | 86D06 | 1.00 | 0.50 | 0.7 | 20.8 | 0.054 | 0.148 |
|  | 8PZ | 3.00 | 1.00 | 0.5 | 10.9 | 0.005 | 0.192 |
|  | 8V5 | 0.00 | 0.50 | 0.3 | 5.5 | 0.046 | 0.242 |
|  | E6C | 2.50 | 3.00 | 19.4 | 67.3 | 0.109 | 0.053 |
|  | F436 | 1.00 | 0.50 | 2.6 | 20.4 | 0.002 | 0.170 |
|  | F754 | 0.50 | 0.50 | 2.0 | 27.4 | 0.088 | 0.200 |
|  | T4C | 2.00 | 1.00 | 2.8 | 22.3 | 0.007 | 0.189 |

| **Corpus**  Time | ID | Gastritis  score | *H.pylori*  density  score | % sLe^x^ staining in surface | % sLe^a^ staining in surface |
| --- | --- | --- | --- | --- | --- |
| Pre-  Inoculation | 82A49 | 0.50 | 0.00 | 2.4 | 56.5 |
|  | 85D08 | 0.50 | 0.00 | 0.5 | 0.5 |
|  | 86D02 | 0.00 | 0.00 | 24.2 | 62.6 |
|  | 86D06 | 0.00 | 0.00 |  |  |
|  | 8PZ | 0.50 | 0.00 | 13.1 | 21.7 |
|  | 8V5 | 0.00 | 0.00 | 0.4 | 27.1 |
|  | E6C | 0.00 | 0.00 | 0.0 | 26.7 |
|  | F436 | 0.00 | 0.00 | 48.5 | 56.3 |
|  | F754 | 0.00 | 0.00 | 20.5 | 28.5 |
|  | T4C | 0.00 | 0.00 | 8.1 | 20.3 |
| 1 week | 82A49 | 2.00 | 1.00 | 9.6 | 91.0 |
|  | 85D08 | 2.00 | 0.50 | 4.4 | 55.4 |
|  | 86D02 | 0.00 | 0.00 | 24.4 | 25.9 |
|  | 86D06 | 0.00 | 0.00 | 0.0 |  |
|  | 8PZ | 0.50 | 0.50 | 0.5 | 59.6 |
|  | 8V5 | 0.00 | 1.00 | 0.0 | 38.0 |
|  | E6C | 1.50 | 0.50 | 0.7 | 18.5 |
|  | F436 | 1.00 | 0.00 | 7.6 | 85.8 |
|  | F754 | 0.00 | 0.00 | 8.8 | 57.9 |
|  | T4C | 0.00 | 0.00 | 18.4 | 51.1 |
| 2 months | 82A49 | 1.00 | 0.00 | 16.1 | 80.9 |
|  | 85D08 | 2.25 | 0.50 | 7.4 | 45.5 |
|  | 86D02 | 1.00 | 0.00 | 21.2 | 44.5 |
|  | 86D06 | 1.00 | 0.00 | 26.2 | 67.1 |
|  | 8PZ | 1.50 | 0.50 | 4.8 | 72.1 |
|  | 8V5 | 0.00 | 0.00 | 3.1 | 76.1 |
|  | E6C | 1.50 | 0.00 | 0.7 | 52.6 |
|  | F436 | 0.00 | 0.00 | 5.4 | 55.7 |
|  | F754 | 0.00 | 0.00 | 8.2 | 72.7 |
|  | T4C | 0.00 | 0.00 | 23.6 | 52.9 |
| 4 months | 82A49 | 1.00 | 0.00 | 0.0 | 84.1 |
|  | 85D08 | 2.00 | 2.00 | 0.1 | 22.0 |
|  | 86D02 | 0.00 | 0.00 | 7.6 | 44.8 |
|  | 86D06 | 1.00 | 0.00 | 1.0 |  |
|  | 8PZ | 1.00 | 0.50 | 0.3 | 53.3 |
|  | 8V5 | 0.00 | 0.00 | 0.5 | 14.9 |
|  | E6C | 1.50 | 1.00 | 0.0 | 28.2 |
|  | F436 | 0.00 | 0.00 | 5.9 | 80.8 |
|  | F754 | 0.00 | 0.00 | 52.6 | 56.6 |
|  | T4C | 0.00 | 0.00 | 3.9 | 29.7 |
| 7 months | 82A49 | 2.00 | 0.50 | 0.0 | 87.8 |
|  | 85D08 | 2.00 | 3.00 | 0.0 | 4.9 |
|  | 86D02 | 0.00 | 0.00 | 14.7 | 45.3 |
|  | 86D06 | 1.00 | 1.00 | 1.3 | 36.6 |
|  | 8PZ | 2.00 | 1.00 | 0.0 | 64.9 |
|  | 8V5 | 0.00 | 0.00 | 0.9 | 37.6 |
|  | E6C | 2.00 | 1.00 | 25.4 | 57.6 |
|  | F436 | 0.00 | 1.00 | 2.3 | 26.7 |
|  | F754 | 0.00 | 2.00 | 24.1 | 46.7 |
|  | T4C | 0.00 | 0.00 | 11.7 | 47.8 |
| 10 months | 82A49 | 2.00 | 1.00 | 0.0 | 90.2 |
|  | 85D08 | 3.00 | 2.00 |  |  |
|  | 86D02 | 0.00 | 0.00 | 13.2 | 60.1 |
|  | 86D06 | 1.00 | 1.00 | 0.0 | 15.4 |
|  | 8PZ | 2.00 | 0.00 | 0.2 | 22.6 |
|  | 8V5 | 0.00 | 0.00 | 0.1 | 26.9 |
|  | E6C | 1.00 | 1.00 | 1.6 | 5.8 |
|  | F436 | 0.50 | 1.00 | 18.6 | 64.7 |
|  | F754 | 0.00 | 0.00 | 16.9 | 26.7 |
|  | T4C | 1.00 | 0.00 | 3.4 | 59.4 |
